# Reply to ‘Cost-effectiveness of long term left ventricular assist devices’

**DOI:** 10.1007/s12471-024-01910-7

**Published:** 2024-11-05

**Authors:** Isabell Wiethoff, Birgit Goversen, Michelle Michels, Jolanda van der Velden, Mickaël Hiligsmann, Tom Kugener, Silvia Evers

**Affiliations:** 1 Department of Health Services Research, Care and Public Health Research Institute (CAPHRI), Maastricht University, Maastricht, The Netherlands; 2 Department of Physiology, Amsterdam UMC, Vrije Universiteit, Amsterdam Cardiovascular Sciences Institute, Amsterdam, The Netherlands; 3 Department of Cardiology, Thorax Center, Cardiovascular Institute, Erasmus MC Rotterdam, Rotterdam, The Netherlands; 4 Netherlands Institute of Mental Health and Addiction, Centre for Economic Evaluation, Trimbos Institute, Utrecht, The Netherlands

We appreciate the interest in our manuscript and the growing attention on cost-effectiveness research.

Van Hout et al. refer to our graphical abstract, which functions as a structural approach to summarize existing economic evaluations for inheritable cardiomyopathies published until April 2021. Thereby, we simply described each study’s conclusion on the cost-effectiveness of a given intervention without including any personal reflections. As the figure itself did indeed not assess each study’s generalizability and transferability, we highlighted the number of included studies per category and concluded, in the graphical abstract as well as in the main text, that more research is needed to guide decision-making [1].

With great interest we read the study by Lim et al. (2022) saying that LVAD therapy may be cost-effective in advanced heart failure patients, which falls beyond the scope of our review as it was published after April 2021 and did not specifically target cardiomyopathy patients [2]. In the future, updated versions of our systematic review will be essential to capture emerging evidence and ensure the continued relevance of the findings.
